# Combining Sensors Information to Enhance Pneumatic Grippers Performance

**DOI:** 10.3390/s21155020

**Published:** 2021-07-24

**Authors:** Rocco Antonio Romeo, Michele Gesino, Marco Maggiali, Luca Fiorio

**Affiliations:** Istituto Italiano di Tecnologia, iCub-Tech Facility, Via San Quirico 19d, 16163 Genoa, Italy; michele.gesino@iit.it (M.G.); marco.maggiali@iit.it (M.M.); luca.fiorio@iit.it (L.F.)

**Keywords:** pneumatic grippers, force sensor, torque sensor, proximity sensor, center of pressure, state machine

## Abstract

The gripper is the far end of a robotic arm. It is responsible for the contacts between the robot itself and all the items present in a work space, or even in a social space. Therefore, to provide grippers with intelligent behaviors is fundamental, especially when the robot has to interact with human beings. As shown in this article, we built an instrumented pneumatic gripper prototype that relies on different sensors’ information. Thanks to such information, the gripper prototype was able to detect the position of a given object in order to grasp it, to safely keep it between its fingers and to avoid slipping in the case of any object movement, even very small. The gripper performance was evaluated by means of a generic grasping algorithm for robotic grippers, implemented in the form of a state machine. Several slip tests were carried out on the pneumatic gripper, which showed a very fast response time and high reliability. Objects of various size, shape and hardness were employed to reproduce different grasping scenarios. We demonstrate that, through the use of force, torque, center of pressure and proximity information, the behavior of the developed pneumatic gripper prototype outperforms the one of the traditional pneumatic gripping devices.

## 1. Introduction

Robotics is spreading in multiple domains. One of the key capabilities of robots is the possibility to grasp and manipulate objects of various sizes and shapes. This can be accomplished by resorting to robotic grippers, which can be viewed as the “hands” of the robot. Lately, robotic grippers started evolving from pick-and-place tools to more complex devices, able to carry out difficult operations. The employment of sensors and the development of reliable algorithms allows the gripper to achieve “smart” capabilities.

One of such capabilities is that of slip avoidance [1]. To securely grasp an object, the gripper must not only grip it but also promptly react if slip takes place [2]. Moreover, the gripper has to “understand” when to close (or open) its fingers, e.g., when it has to pick (or release) a given object. To add useful information, vision and/or proximity sensors should be integrated in the gripping systems. Such sensors provide the gripper with knowledge about the object position so as to grasp it when the best conditions apply. In this concern, one of the first works is [3], where a robotic gripper was endowed with proximity sensors on both palm and fingers. To control the grasping force, a center-of-pressure (CoP) sensor was adopted. Although it was possible to manage the position of the object to be grasped, the force information was missing and only the CoP sensor output, in (V), was available. Moreover, grippers were also provided with opto-mechanical tactile sensors to improve force adjustment [4], though no detail about the object position was involved before the grasping action.

Generally, electric grippers are more often endowed with torque and/or and position sensors that permit to adjust the exerted force and the fingers’ position. For instance, in [5], an electric gripper was used which featured capacitive pressure sensors to ensure grasp stability. Electric, soft grippers are also gaining attention: haptic feedback represents a valid solution for achieving sensorization [6].

Despite being more difficult to control, pneumatic grippers are the most common ones in the industry [7]. However, such grippers usually execute only open–close operations without controlling the applied force [6]. In most applications, pneumatic grippers’ sensorization is rather poor. Some attempts were performed which mainly took into account force sensors. This is true for both rigid [7,8] and soft [9] pneumatic grippers. Furthermore, accelerometers were mounted on pneumatic grippers to estimate the grasped object hardness [10] and recently, the object shape [11]. Nevertheless, the collaboration with humans was not involved; the scarce capability to interact with the surrounding environment constitutes one of the biggest limitations of robotic grippers [12].

**Figure 1:**
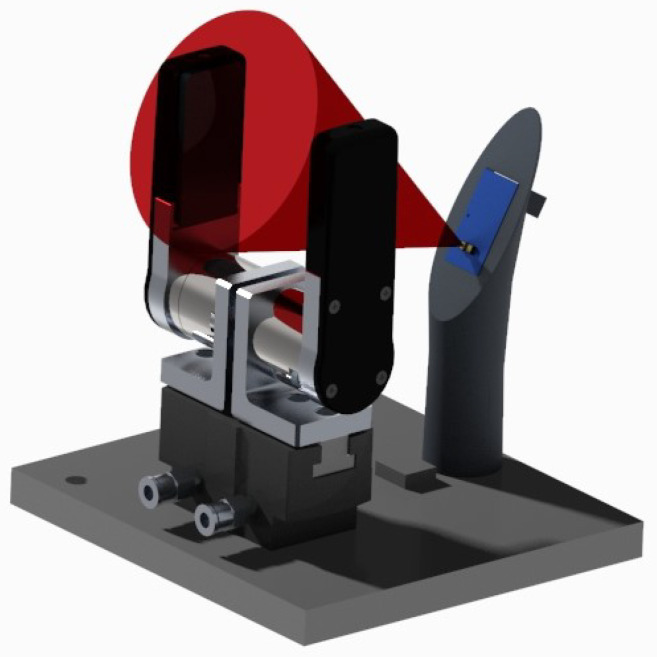
CAD rendering of the experimental setup. The red cone represents the proximity sensor field of view.

The present article was motivated by the intention to enhance the performance of pneumatic grippers. To this aim, we created a specific setup composed of an instrumented pneumatic gripper prototype that could resort to different sensors’ output. The prototype was actuated by means of two external pressure regulators, and was equipped with custom-designed fingers and with a six-axis, force/torque (F/T) sensor which served to measure the grip force and to retrieve the CoP components. An IR-based proximity sensor (PS) with conic field of view was mounted on a support which was in turn located close to the gripper, as illustrated in the rendering of Figure 1. This sensor was utilized to detect the object presence and its positioning before grasping it.

To demonstrate the improvement with respect to (w.r.t.) state-of-the-art pneumatic grippers, we elaborated and applied on our prototype a simple algorithm that can be used on collaborative grippers in general. Through the implementation of such an algorithm, we show the following capabilities of the pneumatic gripper prototype to: (I) recognize whether there is an object to be grasped between the gripper fingers; (II) recognize when the object is in proper position for grasping; (III) recognize when the grasped object is correctly maintained between the gripper fingers; and (IV) recognize whether the grasped object is moving, hence preventing it from slipping off.

It is worth highlighting that, to the best of the authors’ knowledge, this is the first tentative to combine force, torque, CoP and proximity information using a rigid pneumatic gripper. The same applies to the functionalities that we were able to achieve. Hence, instead of the traditional fully open/fully closed behavior, our pneumatic gripper prototype might operate in a smart, collaborative way, from checking the presence of an object between its fingers, therefore before starting the grasping operation, to the completion of the grasping operation itself. As the object position is considered correct, the gripper is commanded to grasp it. Then, as the grasp is deemed as stable, a reference position is computed and during the grasping, every object movement w.r.t. such a reference position will be compensated by increasing the grasping force.

To this end, a closed-loop force control algorithm, previously developed by the authors, was employed [7]. The slip detection is performed by means of the CoP evaluation; however, in our case, the CoP is estimated through the F/T sensor without requiring a specific sensor as, e.g., in [3,13]. In contrast to other instrumented grippers, such as the one of [14], no additional sensors are needed for slip detection.

As regards proximity sensors, their presence somehow introduces a visual feedback to be used together with tactile and force sensing, creating a sort of continuous sensory modality [15] from which robotics’ applications might strongly benefit. The integration of proximity sensors in robotic fingers was already performed in precedent works such as [16]. Another effective solution to obtain reliable vision resorts to costly and bulky sensors. Consider, e.g., [17] where an RGB-D camera was used to capture the initial grasp pose before a robotic gripper could carry out some tasks achieving stable grasp and slip avoidance. Cameras might be adopted to detect the presence and the contour of an object to be grasped by the gripper as in [18], or else the surface deformation map of a grasped object [19]. Alternatively, we employ a small, cheap proximity sensor as our intention is currently limited to prove the prototype ability to recognize the object position. This is enough to allow the operator to place the object between the gripper fingers in a collaborative manner, awaiting a quick, automatic response by the gripper itself.

Fifty experiments were conducted to assess the pneumatic gripper prototype behavior. Five objects of diverse size, shape and hardness were tested, performing as many experiments per object and per type of slip (i.e., linear and rotational).

The remainder of the article contains the following sections: Section 2 describes the pneumatic gripper prototype and the experimental setup, whereas Section 3 illustrates the implemented algorithm; Section 4 shows the results and discusses them, finally Section 5 summarizes the conclusion and future work.

## 2. Materials and Methods

In this Section, it will be described the pneumatic gripper prototype and how it was actuated and instrumented. Furthermore, we will present the experimental setup.

### 2.1. Gripper Prototype

The built pneumatic gripper prototype is based on a commercial pneumatic one, i.e., CGPT20 by Camozzi Automation. It features a pneumatic piston actuated by the air injected inside the gripper chambers. The air pressure in such chambers was controlled by means of two external regulators: one for the opening and one for the closing chamber. The piston relayed its force and displacement to the jaws through two levers, resulting in the jaws’ movement. The levers were characterized by a rotational movement, whereas the jaws, and the piston as well, moved linearly. The gripper stroke was 4 mm, and its grasp force ranges from 0 to 110 N ca. More details about the gripper and the pneumatic actuation can be retrieved in [7].

**Figure 2:**
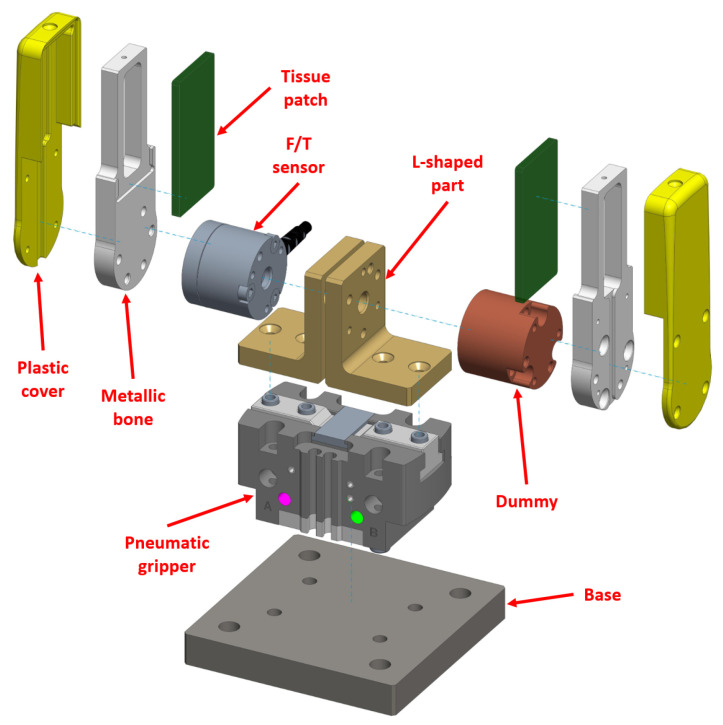
Exploded view of the instrumented prototype. The pneumatic gripper mounted on the base was the commercial device (CGPT20) upon which the whole instrumented prototype was built.

Now, such a gripper came without fingers and sensors of any kind. Therefore, we created our prototype (Figure 2, exploded view) by mounting some 3D-designed parts and some off-the-shelf sensors. A similar prototype proved to be ideal for testing and validating the smart functionalities mentioned in the Introduction by means of the algorithm that will be presented later.

As in Figure 2, the F/T sensor was interfaced on one side to a L-shaped part which was mounted directly above the gripper right jaw. On the other side, the F/T was connected to another part acting as a finger. Such a finger was composed by an inner, metallic core (i.e., bone) and by a plastic cover facing outwards. A soft tissue patch made of Neoprene was instead attached on the internal face of the finger.

The left part of the prototype was identical with the exception of the F/T, which this time was replaced by a metallic cylinder (dummy). The latter, as well as the L-shaped parts and the finger components, were designed using the software Creo Parametric, then machined and assembled.

**Figure 3:**
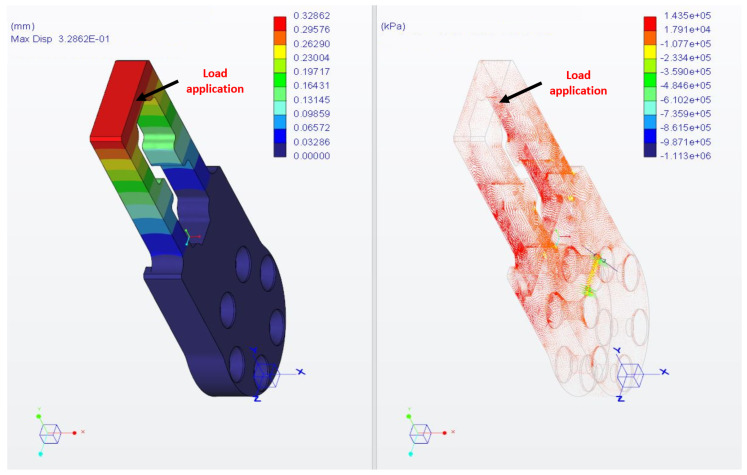
Results of the FEM analysis on the metallic finger simulating the application of a 100-N load on the top. On the right, the principal stresses are shown, whereas on the left, the structure displacement is depicted. The software used for the simulations was Creo Parametric.

The material choice for the finger was validated with structural FEM calculations. To simulate the heaviest operating condition, a load of 100 N was applied in a small surface region at the top extremity of the finger, as depicted in Figure 3, with proper constraints set in the correspondence of the screw holes (not shown). The chosen material for the finger, as well as for the L-shaped part and for the dummy, is Ergal 7075-T6. It is characterized by a minimum yield strength σ of 430 MPa. The results of the FEM calculations are illustrated in terms of displacement and principal stresses in Figure 3, in the left plot and right plot, respectively.

The maximum strains (143 MPa) are located near the fixing screw holes, but these can be viewed as singular calculation points. Instead, the critical loads of interest are located at the extremity of the finger near the load application area. Their maximum value *M* is 130 MPa maximum, resulting in a security factor S=σ/M higher than 3. Such a result is definitely adequate, considering that the maximum force level in the experiments will be as high as 30 N.

As regards the maximum displacement, it is located at the load application area, as expected, and has an acceptable value of 0.32 mm.

### 2.2. Experimental Setup

**Figure 4:**
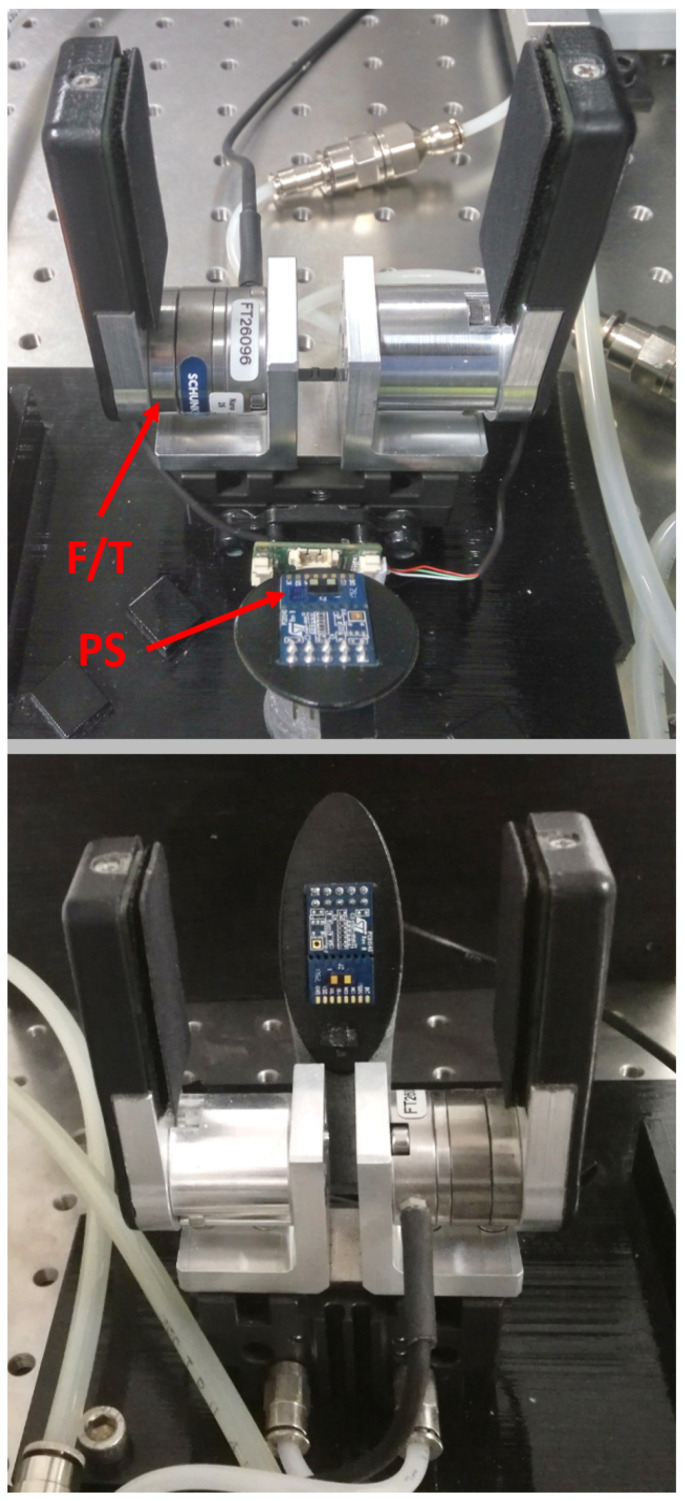
Smart gripper prototype: (**top**) rear view; and (**bottom**) front view.

In addition to the gripper prototype, the experimental setup includes a PS located on a dedicated support, as illustrated in Figure 4. The PS support was 3D-printed as well. In the current setup, which constitutes our first attempt to provide a pneumatic gripper with multiple sensors information, the PS was not integrated but was rather located externally. We are developing a fully integrated pneumatic gripper prototype embedding more PSs without external supports. Such an integrated prototype will be presented in future works, as is also reported in the Conclusion.

The pressure regulators (not shown) actuating the gripper were positioned very close to it, so as to guarantee minimum pressure loss and delay. The regulators could yield a maximum pressure as high as 10 Bar.

All the sensor data (with the exception of the PS) were acquired at a sampling frequency of 1 kHz, and the pressure regulators actuating the gripper were piloted at the same frequency. To this aim, a connector block, namely NI SCB 68A, was interfaced to a NI 6251 data acquisition device. The controller employed to drive the pressure regulators and to adjust the gripper grasping force was the one developed by the authors in [7].

The F/T sensor was the Nano 25 by Ati Industrial Automation, Inc. It had a force resolution of 1/8 N on the grasping force direction, and 1/660 Nm for the torques in the orthogonal directions. This sensor can measure forces and torques up to 1000 N and 6 Nm, respectively.

The PS was the VL53L1X, which can sense in the range of [4–40] mm with a 1 mm resolution. Its output was acquired through a STM32F401RE-Nucleo board at 10 Hz. The sensor output included: the distance (pos), its standard deviation (SD) and the ambient light (AL). Both the PS and the acquisition board were manufactured by STMicroelectronics.

The whole software architecture was developed in a LabVIEW environment.

## 3. Algorithm Description

To test the gripper prototype capabilities, we conceived an algorithm that might be applied to any gripping system composed of a gripper body and two fingers. The execution of such an algorithm permits to observe the gripper behavior through the entire grasping action, also recognizing the correct position of the object in order to grasp it; moreover, it allows the gripper to close its fingers reaching a stable grasp, and then to carry out an anti-slip action in case of contact loss. The algorithm resorts to the information obtained by means of a PS, which measures the position of the object being grasped; by means of a grasping force sensor (FS), namely measured grasping force ($F_{M}$); and by means of a center-of-pressure sensor (CoPS), namely CoP within the gripper fingers. Given our prototype configuration, the F/T sensor simultaneously acts as FS and CoPS.

The proposed algorithm can be schematized as a state machine. That is, it is composed of a sequence of states, during which the system (i.e., the gripper) carries out one or more operations. A set of transitions defines how to pass from one state to the other. Each transition occurs when one or more sensory inputs satisfy some pre-defined conditions. The following definitions have to be considered before explaining our state machine:

- ***F*_1_, *F*_2_**: desired values of grasping force;
- ***ThF***: threshold of measured force to discern a stable grasp;
- ***ThPos***: threshold of PS measurement, i.e., pos;
- ***ThSD*, *ThAL***: thresholds of PS measurement, i.e., SD and AL;
- ***ThCoP***: threshold of CoPS measurement.

In our case, the state machine includes the following states:

- **State 1**—IDLE. The gripper fingers are open and no operation is performed. The PS constantly monitors the grasping area to detect objects to be grasped;
- **State 2**—GRASP. The controller generates the desired force level $F_{1}$. The gripper grasps the object but no stable grasp is achieved yet, i.e., $|F_{1}-F_{M}|>ThF$;
- **State 3**—COMPUTE ZERO. The zero position is being computed;
- **State 4**—HOLD. The gripper grasps the object and checks if the grasp is stable;
- **State 5**—TIGHTEN. The desired force level $F_{2}$ is generated by the controller. The gripper increases the force exerted on the object to avoid slip.

In addition, the transitions are described below:

- **Transition 1**—OBJECT DETECTED. The object position, between the gripper fingers, is under a certain threshold such that Pos<ThPos. To ensure stability, the *SD* of the quantity pos must be under a threshold *ThSD* and the *AL* must be under a threshold *ThAL*. Therefore, the object will be correctly detected if SD<ThSD and AL<ThAL also hold true;
- **Transition 2**—STABLE GRASP. The grasp force is deemed stable, i.e., $|F_{1}-F_{M}|<ThF$ for a certain time window (e.g., 0.5 s);
- **Transition 3**—ZERO COMPUTED. The Zero position (Zero) is computed as the grasp force is stable. The zero position can be the average of a number of samples from the CoPS, collected, e.g., for 1 s;
- **Transition 4**—OBJECT MOTION DETECTED. A movement of the grasped object is detected, i.e., (||CoP||−Zero)>ThCoP. That is, the norm CoPN=||CoP|| is too far from the reference position;
- **Transition 5**—RELEASE REQUESTED. The gripper is requested to release the object. This happens when the grasp operation is over. This condition may also occur without involving State 5, i.e., whether the grasp operation will be completed without detecting object motion.

**Figure 5:**
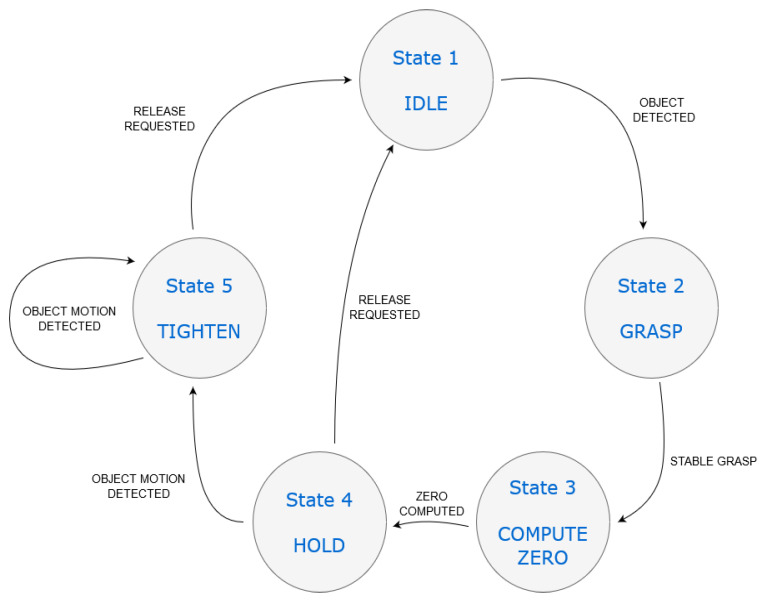
Representation of the state machine.

A graphical representation of the state machine is given in Figure 5. Note that the transition RELEASE REQUESTED may happen from either State 4 (HOLD) or State 5 (TIGHTEN) to State 1 (IDLE). This derives from the fact that a slip phenomenon will not necessarily occur. As a consequence, if State 4 will be successful, it will also be the last before returning to State 1.

**Figure 6:**
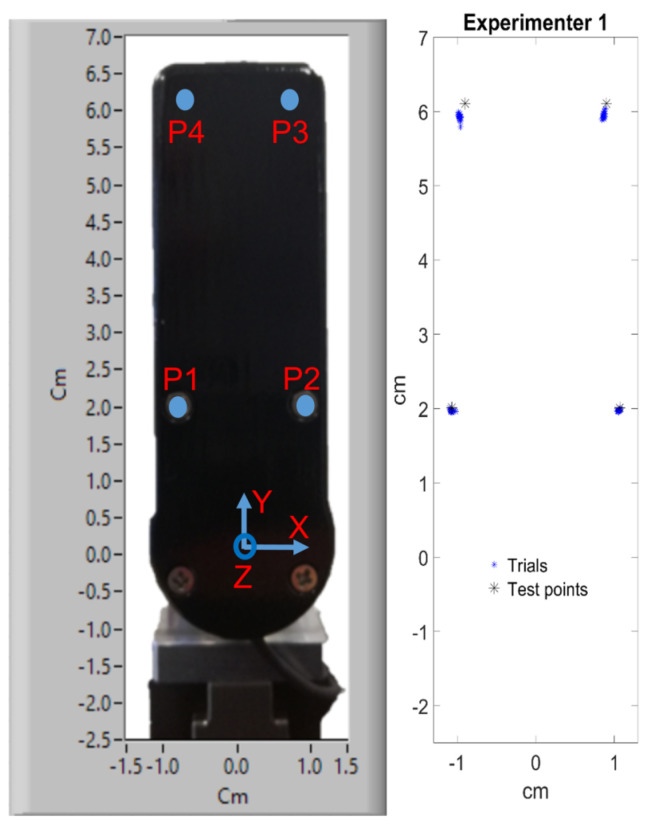
CoP estimation on the 4 predefined points. On the **left**, real finger with the coordinate frame and points. On the **right**, the cloud of reconstructed points (blue) and actual points (black asterisks).

The components of the CoP were retrieved by resorting to the F/T measurements. In more detail, three components were needed to estimate the CoP position on both the axes of the finger surface. The following relations were considered:

(1) $$\left\{\begin{array}{c} CoP_{X}=\frac{T_{Y}}{F_{Z}}\\ CoP_{Y}=\frac{-T_{X}}{F_{Z}} \end{array}\right.$$

in which the axes are oriented as in Figure 6. The quantity $F_{M}$ coincides with the force $F_{Z}$. Since the force $F_{Z}$ is always positive during the grasp, the sign of the CoP components may vary only depending on the torques sign. Therefore, such a sign is set accordingly in Equation (1) for both $T_{X}$ and $T_{Y}$ to achieve correct CoP variation. More details will be provided in the next Section. Finally, the norm CoPN of the vector containing $CoP_{X}$ and $CoP_{Y}$ is calculated.

## 4. Experiments and Results

This Section will illustrate the experiments performed on the setup described in Section 2, and will discuss their results.

### 4.1. Experiments

**Figure 7:**
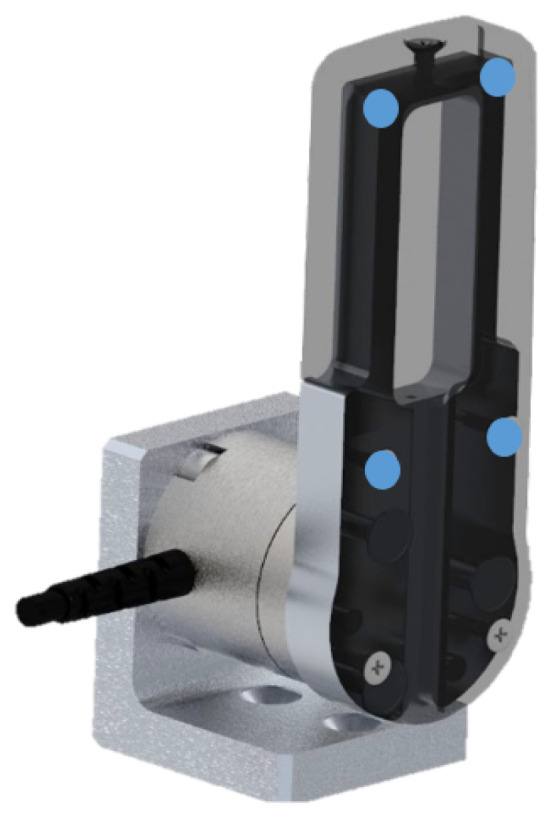
Detail of the gripper custom finger with plastic cover (set transparent), inner metal structure, L-shaped part and F/T sensor. The 4 points for validating the CoP estimation are shown as well.

Before proceeding with the gripper performance assessment, the validation of the CoP estimation will be shown. Two experiments were performed by as many operators as possible (namely Exp1 and Exp2), during which four predefined points were manually pressed by means of a thin probe. Each point was pressed 10 times. The finger cover was removed and the experiments were performed on the inner metallic structure, where the points were easily recognizable (see Figure 7).

The PS was also calibrated according to the sensor guide provided by the manufacturer. Offset and crosstalk were retrieved following the suggested procedure. The sensor fundamental parameters were chosen after an experimental evaluation.

To validate our prototype capabilities, we performed 50 experiments reproducing two different slip conditions. The gripper was required to grasp five objects of different shape, size and hardness: once the object was stably grasped (HOLD state), slip was manually induced by an operator by either pulling the object upwards (linear) or pushing it on its top (rotational). Therefore, the gripper performance was assessed in terms of:

- Correct object position recognition;
- Proper object grasp;
- Prompt slip avoidance.

All the thresholds mentioned thus far were empirically determined. In more detail, ThF was chosen to be equal to 1.5 N, guaranteeing that the stable grasp could exist only if the measured force was very close to the desired one. ThCoP was 0.2 cm, enough small to detect even a very limited movement of the grasped object. Depending on the application, if the sensitivity of slip detection can be lower, this threshold may be higher. ThPos was 7 cm, a value that allowed a proper grasp for all the test objects. When selecting this value, the minimum sensing distance, which is 4 cm according to the PS datasheet, had to be taken into account. As regards ThSD, a value of 0.125 cm was found to be satisfactory. Finally, the ThAL was set to 0.15 mega counts per second (MCPS) in order to reduce the light interference due to the reflection from the environment. The last two thresholds were particularly important to obtain a stable PS signal, with the lowest possible fluctuations. As also reported in the next subsection, when SD and AL were above their thresholds, the pos signal was assigned a NaN value.

For what concerns the force level $F_{1}$, it was set to 7.5 N, being this value sufficient to grasp all the objects. $F_{2}$ was four times higher than $F_{1}$, i.e., 30 N, to generate a considerable grip force increment in case of slip. However, for the two softest objects, i.e., soft cylinder and styrofoam piece, $F_{2}$ was 20 N.

**Figure 8:**
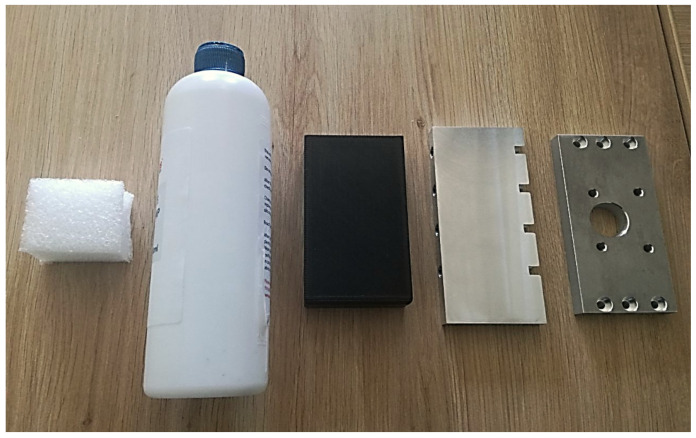
The five objects employed during the experiments. From left to right: styrofoam piece; soft cylinder; rigid plastic piece; metal piece; holed metal piece.

The five tested objects, illustrated in Figure 8, were: two rectangular metal pieces, one of which had holes potentially disturbing the infra-red-based PS; one rigid plastic piece; one soft cylinder; and one styrofoam piece. In this way, rather different grasping conditions were assured. Indeed, this set of objects includes items with diverse length as the soft cylinder is six times longer than the styrofoam piece. Furthermore, the objects ranged from very rigid ones (metallic parts) to very soft ones (styrofoam and soft cylinder), regardless of the stiffness. The heaviest objects had a mass of 0.2 kg (metallic pieces), however, heavier objects could be employed as the maximum grasp force of the gripper can be higher than 100 N. The only constraint that must be taken into account is the object size. Due to the limited gripper stroke, i.e., 4 mm, we had to select objects with very a similar size. The distance between the fingers’ surface is 55 mm, permitting to grasp even larger objects despite the limited stroke.

### 4.2. Results

**Figure 9:**
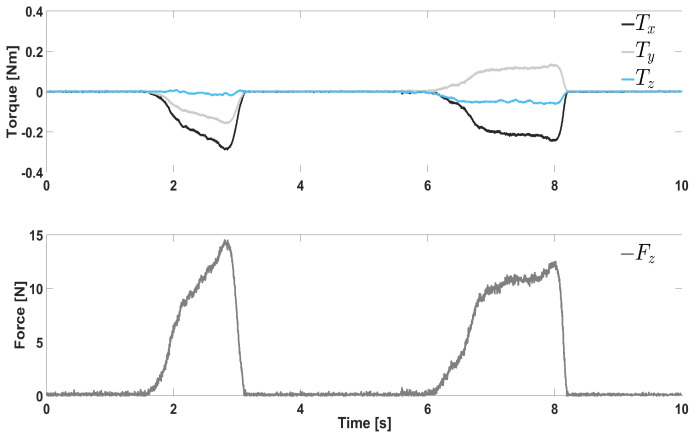
Normal force and torques measured by the F/T when pressing on P1 (**left**) and P2 (**right**).

First, we validated the formulas used to estimate the CoP, i.e., Equation (1). Figure 9 plots the measured torques and the force $F_{Z}$ when the operator applied a pressure with the aforementioned probe on P1 and P2 (according to Figure 6). Clearly, the torque $T_{X}$ always has the same sign, i.e., negative, as both the application points P1 and P2 are above the F/T sensor reference frame. Therefore, it is convenient to use the negative value $-T_{X}$ to achieve a positive coordinate $CoP_{Y}$. On the other hand, the torque $T_{Y}$ changes sign depending on where the pressure is exerted. Specifically, P1 and P2 are located on opposite sides w.r.t. the reference frame origin, yielding negative (P1) and positive (P2) $T_{X}$ values. As a consequence, the sign of $T_{Y}$ must be taken into account when computing $CoP_{X}$, avoiding to adopt the torque absolute value. In this way, the $CoP_{X}$ coordinate will have a correct sign, being positive for P1 and a negative for P2. According to Equation (1), very little force Fz will generate uncertainty. To eliminate such uncertainty, both $CoP_{X}$ and $CoP_{Y}$ were set to be null whenever the grasping force Fz was lower than 1 N.

Figure 6 shows the results of the CoP estimation by means of Equation (1). The four selected points are shown on the finger with cover, even though the experiments were done on the bare metallic bone. The results summarized in Table 1 show the average ±—the standard deviation on the 10 trials executed on each point. Very high accuracy was achieved, with the maximum error equal to 2 mm, i.e., ca. 3% (P4 y coordinate, Exp1). Such results confirmed that the CoP was correctly estimated through Equation (1) and that the algorithm could rely on it. Consider that the maximum full-scale error of the employed F/T sensor is ±10 N for $F_{Z}$, resulting in very high theoretical errors (several cms) at low forces/torques as the ones in Figure 9.

**Figure 10:**
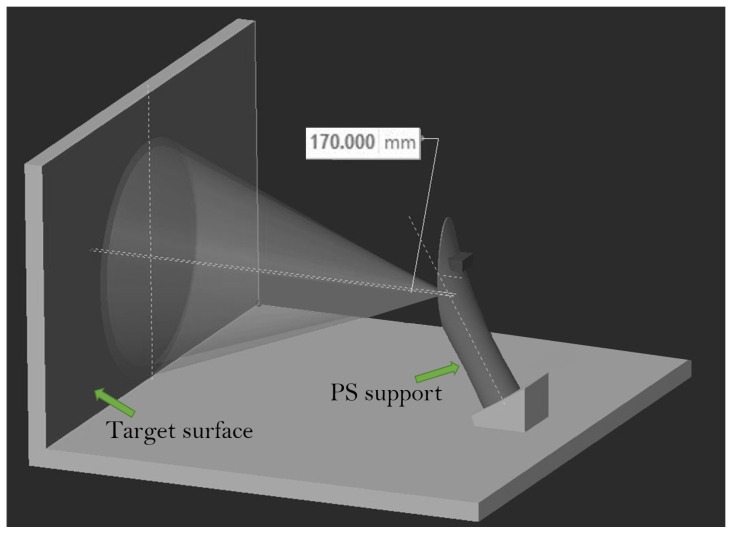
PS calibration setup.

Concerning the PS sensor, its calibration for both offset and crosstalk was carried out according to the manufacturer indications. To this end, a specific setup (Figure 10) was constructed; the PS was mounted on its support as in Figure 4, and located in front of a target surface. The support was in turn inclined in such a way that the PS was parallel to the target surface. We do not report here the procedure, which is available on the web. We report instead our analysis about some fundamental parameters of the PS, namely time budget (TB) and the inter-measurement period (IMP). The first indicates how much time the sensor spends doing a single measurement, whereas the second is the actual interval between one measurement and the subsequent. It is intuitive that the higher the TB is, the higher the power consumption is, as well as the sensor precision. However, it is the IMP that determines the actual measurement frequency of the sensor. These parameters influence the performance of the chosen PS and have to be properly selected. To achieve higher frequencies with good precision, it is reasonable to set both parameters so that TB=IMP−γ, where γ is as little as possible, usually 5 ms.

**Figure 11:**
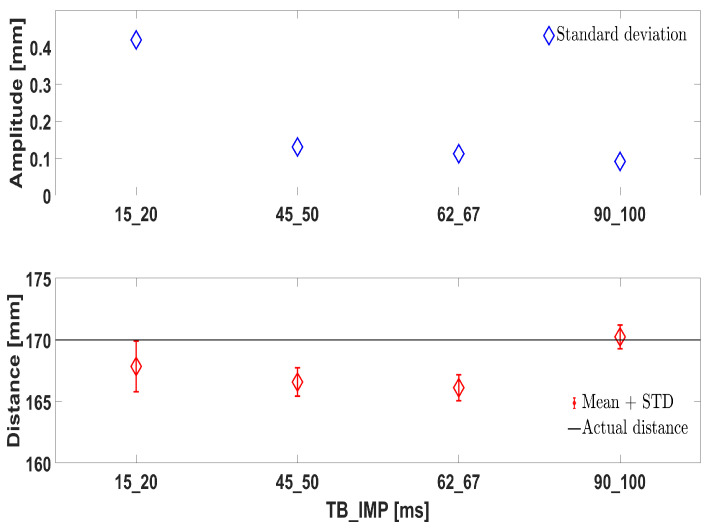
Couples of TB and IMP tested and the consequent PS performance.

From Figure 11, when TB and IMP are too short, i.e., TB=15 ms and IMP=20 ms, the SD of the PS measurement is too high (top subplot). This leads to low accuracy and introduces measurement uncertainty. On the other hand, a TB of at least 45 ms drastically reduces the SD. That is, a frequency of 20 Hz is the maximum achievable (IMP=50 ms) in order to have a good performance. From the bottom subplot, one can see that the best performance in terms of measurement precision is given with the highest TB and IMP tested. Hence, the couple TB=90 ms and IMP=100 ms was selected. Notice that the distance of 170 mm is recommended by the manufacturer to be used during calibration.

**Figure 12:**
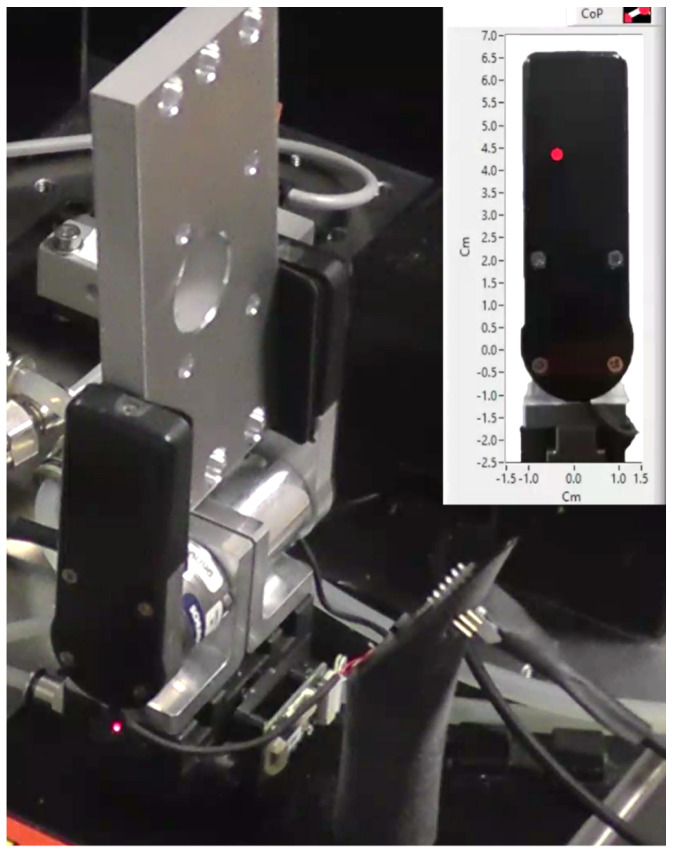
Experiment with the holed metal piece. The red dot represents the estimated CoP.

Figure 12 illustrates the setup during an experiment with the holed metal piece. The state machine was in the HOLD state, maintaining the piece between the fingers and waiting to move either to the TIGHTEN state, in case of movement detection, or else to the IDLE state if the gripper was commanded to reopen. The objects were randomly placed by the human operator between the fingers, in a collaborative fashion, without caring to match a precise grasping position. From the figure, one can see that the metal piece is not exactly aligned along the Y axis; the resulting CoP is located correspondingly. Since the system is in HOLD state, the red dot represents zero. With no perturbation, the grasping operation terminates and the gripper reopens (IDLE). Otherwise, if (||CoP||−Zero)>ThCoP, the TIGHTEN state will be entered. A very small disturbance applied by the operator hand is sufficient to induce the system response.

**Figure 13:**
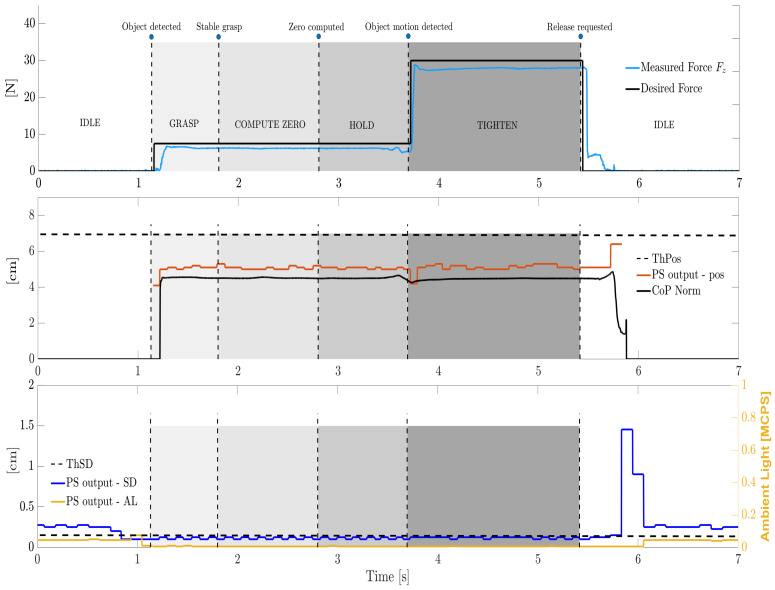
Representative trial on the holed metal piece, linear slip. The **top** subplot shows the desired and grasping forces, whereas the **mid** subplot shows the CoP variation together with the PS distance. The **bottom** subplot shows the AL and SD characterizing the PS signal. For the sake of clarity, labels of transitions and states are reported only in the top subplot whereas only some of the thresholds are plotted.

**Figure 14:**
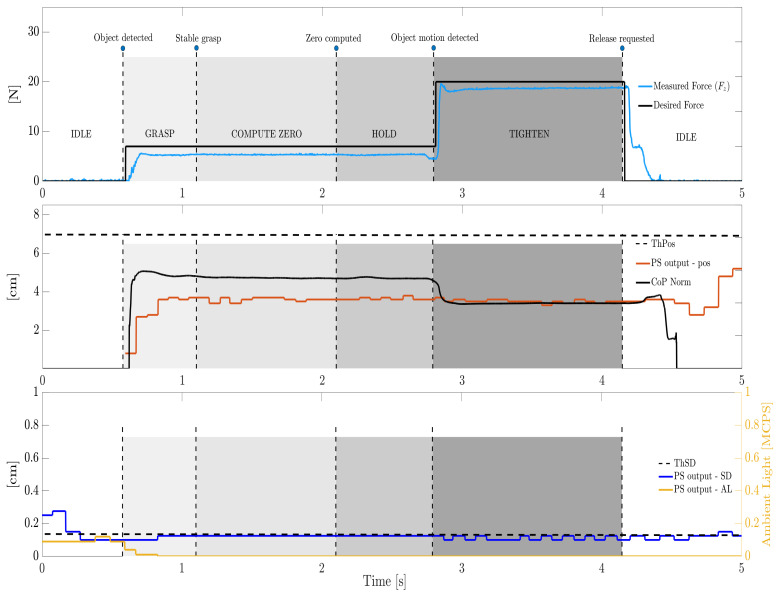
Representative trial on the soft cylinder, rotational slip. The **top** subplot shows the desired and grasping forces, whereas the **mid** subplot shows the CoP variation together with the PS distance. The **bottom** subplot shows the AL and SD characterizing the PS signal. For the sake of clarity, labels of transitions and states are reported only in the top subplot whereas only some of the thresholds are plotted.

Now, we discuss the full experiments with the tested objects. To analyze different grasping conditions due to different geometry and hardness, Figure 13 and Figure 14 illustrate some representative trials with the holed metal piece and with the soft cylinder, respectively. A linear slip was induced to the former, whereas the latter was disturbed with a rotational slip. It must be noticed that the rotational slip is accompanied by a small linear movement, as it is impossible to generate a perfect rotation. Therefore, the gripper will detect the rotational slip thanks to its associated linear component, even though it is very small.

Consider, e.g., Figure 13: until 1 s, both grasping force $F_{Z}$ and the CoP are null, i.e., the gripper is in IDLE state. This can be seen in the first two subplots. In the bottom one, SD and AL are still high, i.e., above their thresholds (not shown). The distance pos fluctuates too much and is regarded as a NaN, where AL and SD not adequate.

As the object approaches the gripper fingers, AL and SD drop below the thresholds ThAL and ThSD, resulting in a valid measurement of pos. This means that the gripper is ready to pass from the IDLE state to the GRASP state. The fingers close as the desired force $F_{D}$ reaches the first level $F_{1}=7.5$ N, typical of the OBJECT DETECTED transition.

Subsequently, $F_{Z}$ quickly grows until $|F_{1}-F_{Z}|<ThF$: as a consequence, the transition STABLE GRASP occurs and the system enters the COMPUTE ZERO state. Here, 1 s is waited to achieve a zero, i.e., a reference value of the norm CoPN=||CoP|| is computed, as it is the mean of all the CoPN values over such a time interval.

As the zero is available, the ZERO COMPUTED transitions bring the system to the HOLD state. At this point (ca. 3.7 s), the experimenter applies a disturbance on the grasped item, inducing a linear slip.

Hence, the OBJECT MOTION DETECTED transition easily takes place, yielding a TIGHTEN state. Therefore, $F_{D}$ reaches the second level $F_{2}=30$ N (as for the other metal piece and for the rigid plastic piece) and the grasping force $F_{Z}$ immediately tracks it. Notice how the CoPN variation generated by the object movement occurs earlier than the pos variation, where the CoP sampling frequency is 100 times higher than the pos one.

Finally, the gripper is commanded to reopen (ca. 5.4 s) through the RELEASE REQUESTED transition, returning in the IDLE state. The object is manually removed as the gripper releases it; the pos signal increases by 1 cm before a NaN is generated again, where SD and AL are again above the thresholds. The CoPN goes towards zero, along with the measured force. A huge peak in the SD signal is evident in the moment when the object is effectively taken by the operator. Such a peak might occur when the object is rapidly removed, provoking an unstable response of the IR-based PS which is suddenly hit by a vast amount of light.

Notice that multiple OBJECT MOTION DETECTED transitions can happen, as more disturbances can be applied. We set the algorithm to make the system react only one time, though there is no theoretical limit.

Most of the above considerations apply to the case of the soft cylinder in Figure 14. For this object, as well as for the styrofoam piece, a lower desired force $F_{2}$ was set, i.e., $F_{2}=20$ N. In contrast to Figure 13, this time the CoPN value is rather higher than the pos one as long as the object is stably grasped. The gripper can manage the object grasping regardless of the CoP location and of the actual object distance measured by the PS (provided that this one falls below its threshold). Interestingly, the PS cannot even catch the rotational slip as no significant variation can be appreciated in the pos signal after the OBJECT MOTION DETECTED transition. The CoPS is instead able to detect the rotational slip as well, which is rather difficult to be observed through a simple proximity sensor.

In terms of overall performance, the success rate was 100% for all the tested objects as far as the following are concerned: (I) object position recognition, as the human operator placed the object between the gripper fingers; (II) grasp stability, as the object was always properly grasped with an adequate measured force level; and (III) slip avoidance, as the disturbance applied by the human operator was all the times compensated by the gripper. Table 2 contains the mean reaction time ReT for all the objects and type of slip, computed as follows:

(2) $$ReT=T_{mov}-T_{re}$$

where $T_{mov}$ corresponds to the time instant when COPN−Zero>ThCoP, i.e., when the slip is actually detected, and $T_{re}$ is the time instant when the measured force $F_{z}$ reached 90% of the desired level F2. Note that at the time $T_{mov}$, the OBJECT MOTION DETECTED transition occurs.

The numbers in Table 2 indicate a very prompt reaction. Apart from the employed force control, which already proved to be effective, such a promptness was possible also thanks to the straightforward actuation of the pneumatic gripper mechanism, which does not feature motors, gears, links, etc. In all the 50 grasping experiments, the reaction time ReT was smaller than 100 ms. This is in line with the human hand reaction time [20], which is also the most dexterous type of gripper in nature [1].

## 5. Conclusions

This work presents an instrumented pneumatic gripper prototype which uses several sensors’ output. The gripper prototype was mounted with custom fingers and resorts to force, torque, CoP and proximity information in an attempt to go beyond the common functioning of traditional pneumatic grippers.

The gripper prototype capabilities were evaluated by implementing a simple algorithm featuring a sequence of operations. A human operator collaboratively placed the object between the gripper fingers. The gripper could recognize whether an object existed between the gripper fingers, as well as the correct object position in order to commence the grasp operation (pick). It also could achieve a stable grasp of such an object, avoiding slip phenomena in case of necessity. The object position was continuously monitored and confronted with a reference one, i.e., the zero position, which is computed as the object is grasped.

Five objects of diverse size, shape and mass were tested. During the grasp, both linear and rotational slip were induced. Results indicated that the gripper prototype was in all the trials able to detect the object position before grasping, to stabilize the grasp and to prevent slip with a fast reaction time. Very small variations of the CoP yielded an appropriate gripper reaction as the desired force was automatically increased.

To the best of the authors’ knowledge, the pneumatic gripper prototype presented in this study constitutes the first attempt to make a pneumatic gripper work with proximity, force, torque and CoP information. The capabilities demonstrated evidently outperform the functioning of the current rigid pneumatic grippers, and allow obtaining a collaborative behavior.

Future steps foresee the testing of a new smart gripper prototype featuring a wider set of sensors. The new gripper is under development and will integrate, among the others, one F/T sensor per finger and two PSs watching between the fingers (the PS is placed on an external support in the present study). Actuation valves were also placed within the gripper structure, avoiding the need for external pressure regulators. In addition, tactile sensor arrays were embedded in the fingers. The new gripper prototype will offer the possibility to implement enhanced versions of our algorithm, including, e.g., an additional control law for managing the slip phenomena instead of performing a discrete compensation through the TIGHTEN state and the OBJECT MOTION DETECTED transition. In addition, more force levels $F_{2}\cdots F_{i}$ can be generated every time a movement is detected; such levels might be pre-defined, or else might obey a proportional trend such that $F_{i}=\alpha F_{i-1}$, where α>1.

Furthermore, the possibility to estimate the stiffness and mass of the grasped object in real time will be evaluated, avoiding having to pre-set different desired force levels for different objects. A greater stroke will allow overcoming the limitation connected to the objects’ size.

## 6. Patents

Based on the content of this article, the following patent application was submitted: “Method of controlling a pneumatic grasping device”, n°IT102021000008006.

## Figures and Tables

**Table 1:** Validation of the CoP estimation (all values in cm).

	P1	P2	P3	P4
x	−1.07	1.07	0.90	−0.90
Exp1 x	−1.07 ± 0.01	1.05 ± 0.01	0.86 ± 0.01	−0.97 ± 0.01
Exp2 x	−1.07 ± 0.02	1.04 ± 0.04	0.87± 0.05	−0.88 ± 0.02
y	1.97	1.97	6.11	6.11
Exp1 y	1.97±0.01	1.97±0.01	5.94±0.04	5.92±0.04
Exp2 y	1.98±0.01	1.97±0.01	6.00±0.04	5.99±0.02

**Table 2:** Reaction time after slip (all values in ms).

	Linear Slip	Rotational Slip
Metal holed piece	41.71 ± 9.37	40.16 ± 3.76
Metal piece	61.5 ± 9.71	52.16 ± 11.83
Rigid plastic piece	54.90 ± 6.77	57 ± 13.38
Soft cylinder	36 ± 5.46	51.14 ± 8.08
Styrofoam piece	71 ± 9.29	64 ± 12.45

## Data Availability

Not applicable.

## References

[1] Romeo R.A., Zollo L. (2020). Methods and Sensors for Slip Detection in Robotics: A Survey. IEEE Access.

[2] Zhang B., Xie Y., Zhou J., Wang K., Zhang Z. (2020). State-of-the-art robotic grippers, grasping and control strategies, as well as their applications in agricultural robots: A review. Comput. Electron. Agric..

[3] Hasegawa H., Mizoguchi Y., Tadakuma K., Ming A., Ishikawa M., Shimojo M. Development of intelligent robot hand using proximity, contact and slip sensing. Proceedings of the 2010 IEEE International Conference on Robotics and Automation.

[4] Saen M., Ito K., Osada K. (2014). Action-intention-based grasp control with fine finger-force adjustment using combined optical-mechanical tactile sensor. IEEE Sens. J..

[5] Romano J.M., Hsiao K., Niemeyer G., Chitta S., Kuchenbecker K.J. (2011). Human-inspired robotic grasp control with tactile sensing. IEEE Trans. Robot..

[6] Chin L., Yuen M.C., Lipton J., Trueba L.H., Kramer-Bottiglio R., Rus D. A simple electric soft robotic gripper with high-deformation haptic feedback. Proceedings of the 2019 International Conference on Robotics and Automation (ICRA).

[7] Romeo R.A., Fiorio L., Avila-Mireles E.J., Cannella F., Metta G., Pucci D. Closed-loop force control of a pneumatic gripper actuated by two pressure regulators. Proceedings of the 2019 IEEE/RSJ International Conference on Intelligent Robots and Systems (IROS).

[8] Romeo R.A., Fiorio L., L’Erario G., Maggiali M., Metta G., Pucci D. (2020). Dynamic Control of a Rigid Pneumatic Gripper. IEEE Robot. Autom. Lett..

[9] Park W., Seo S., Oh J., Bae J. (2020). A Sensorized Hybrid Gripper to Evaluate a Grasping Quality Based on a Largest Minimum Wrench. IEEE Robot. Autom. Lett..

[10] Blanes C., Mellado M., Beltrán P. (2016). Tactile sensing with accelerometers in prehensile grippers for robots. Mechatronics.

[11] Hung C.W., Zeng S.X., Lee C.H., Li W.T. (2021). End-to-End Deep Learning by MCU Implementation: An Intelligent Gripper for Shape Identification. Sensors.

[12] Bi Z., Liu Y., Krider J., Buckland J., Whiteman A., Beachy D., Smith J. Instrumentation of robotic grippers for dynamic control of robotic systems. Proceedings of the 2018 13th IEEE Conference on Industrial Electronics and Applications (ICIEA).

[13] Gunji D., Araki T., Namiki A., Ming A., Shimojo M. (2007). Grasping force control of multi-fingered robot hand based on slip detection using tactile sensor. J. Robot. Soc. Jpn..

[14] Ding Z., Paperno N., Prakash K., Behal A. (2018). An adaptive control-based approach for 1-Click gripping of novel objects using a robotic manipulator. IEEE Trans. Control. Syst. Technol..

[15] Hasegawa S., Yamaguchi N., Okada K., Inaba M. (2020). Online Acquisition of Close-Range Proximity Sensor Models for Precise Object Grasping and Verification. IEEE Robot. Autom. Lett..

[16] Konstantinova J., Stilli A., Althoefer K. Force and proximity fingertip sensor to enhance grasping perception. Proceedings of the 2015 IEEE/RSJ International Conference on Intelligent Robots and Systems (IROS).

[17] Feng Q., Chen Z., Deng J., Gao C., Zhang J., Knoll A. Center-of-Mass-based Robust Grasp Planning for Unknown Objects Using Tactile-Visual Sensors. Proceedings of the 2020 IEEE International Conference on Robotics and Automation (ICRA).

[18] Lévesque F., Sauvet B., Cardou P., Gosselin C. (2018). A model-based scooping grasp for the autonomous picking of unknown objects with a two-fingered gripper. Robot. Auton. Syst..

[19] Zhao K., Li X., Lu C., Lu G., Wang Y. (2017). Video-based slip sensor for multidimensional information detecting in deformable object grasp. Robot. Auton. Syst..

[20] Johansson R.S., Westling G. (1984). Roles of glabrous skin receptors and sensorimotor memory in automatic control of precision grip when lifting rougher or more slippery objects. Exp. Brain Res..

